# Prevalence of Fatigue and Its Association With Quality of Life Among Frontline Clinicians in Ophthalmology and Otolaryngology Departments During the COVID-19 Pandemic

**DOI:** 10.3389/fpsyt.2021.678917

**Published:** 2021-07-09

**Authors:** Yu Jin, Yue Li, Xiu-Ya Li, Yan-Jie Zhao, Teris Cheung, Gabor S. Ungvari, Michael Li, Feng-Rong An, Yu-Tao Xiang

**Affiliations:** ^1^College of Education for the Future, Beijing Normal University, Beijing, China; ^2^Department of Nursing, Beijing Tongren Hospital, Capital Medical University, Beijing, China; ^3^Department of Otorhinolaryngology, Beijing Tongren Hospital, Capital Medical University, Beijing, China; ^4^Unit of Psychiatry, Department of Public Health and Medicinal Administration, Institute of Translational Medicine, Faculty of Health Sciences, University of Macau, Macao, China; ^5^Centre for Cognitive and Brain Sciences, University of Macau, Macao, China; ^6^Institute of Advanced Studies in Humanities and Social Sciences, University of Macau, Macao, China; ^7^School of Nursing, Hong Kong Polytechnic University, Hong Kong, China; ^8^Division of Psychiatry, School of Medicine, University of Western Australia/Graylands Hospital, Perth, WA, Australia; ^9^University of Notre Dame Australia, Fremantle, WA, Australia; ^10^The Hong Kong Academy for Performing Arts, Hong Kong, China; ^11^The National Clinical Research Center for Mental Disorders, Beijing Key Laboratory of Mental Disorders Beijing Anding Hospital, Advanced Innovation Center for Human Brain Protection, School of Mental Health, Capital Medical University, Beijing, China

**Keywords:** COVID-19, fatigue, ophthalmology, otolaryngology, clinicians, quality of life

## Abstract

**Background:** The coronavirus disease 2019 (COVID-19) pandemic has caused psychological distress and heavy burden in medical professionals. This study examined the prevalence of fatigue and its association with quality of life (QOL) in clinicians working in ophthalmology and otolaryngology departments during the COVID-19 pandemic in China.

**Methods:** This was a cross-sectional national online survey conducted between March 15 and March 20, 2020 in China. The severity of fatigue, depression and QOL were measured using the Numeric Rating Scale (NRS), the 9-item Patient Health Questionnaire (PHQ-9), and the World Health Organization Quality of Life Questionnaire-Brief Version (WHOQOL-BREF), respectively.

**Results:** In total, 3,912 clinicians completed the survey (2,155 in ophthalmology department, and 1,757 in otolaryngology department); 2,049 [52.4%; 95% confidence interval (CI) = 50.8–53.9%] reported fatigue (NRS score ≥ 4). Multiple logistic regression analysis revealed that junior clinicians [Odds ratio (OR) = 0.82, 95% CI = 0.68–1.00, *P* = 0.045] had lower risk of fatigue; while clinicians working in tertiary hospitals (OR = 1.23, 95% CI = 1.02–1.49, *P* = 0.029), and the presence of more severe depressive symptoms (PHQ-9 total score ≥ 5; OR = 7.40, 95% CI = 6.29–8.70, *P* < 0.001) were independently associated with higher risk of fatigue. After controlling for covariates, clinicians with fatigue had significantly lower QOL compared with those without [*F*_(1, 3, 911)_ = 283.75, *P* < 0.001].

**Conclusion:** Fatigue was common in clinicians working in ophthalmology and otolaryngology departments during the COVID-19 pandemic. Considering the negative impact of fatigue on clinicians' QOL, health authorities and policymakers should conduct regular screening for fatigue and develop preventive strategies for frontline clinicians working under excessive stress.

## Introduction

The coronavirus disease 2019 (COVID-19) was first reported in China in December 2019 and then it emerged in more than 200 countries and territories (1, 2). COVID-19 is more infectious than Severe Acute Respiratory Syndrome (SARS) and Middle East Respiratory Syndrome (MERS) (3). Hence, the World Health Organization (WHO) declared COVID-19 as a public health emergency of international concern on the January 30, 2020 (1). As of the middle of May 2021, there have been more than 167 million confirmed cases worldwide (4).

The COVID-19 outbreak imposed a heavy burden on health professionals (5) due to long working hours, inadequate personal protective equipment, suboptimal training, shift duty and high-risk of infection (6). Physical and mental health problems were always common in health professionals during outbreaks of infectious diseases. For instance, a systematic review found that 75.9% [95% confidence interval (CI) = 65.9–83.7%] of health professionals during the SARS/MERS/COVID-19 outbreaks reported fever, 41.2% (95% CI = 18.2–33.2%) reported fatigue, 29.0% (95% CI = 14.2–50.3%) reported anxiety and 26.3% (95% CI = 12.5–47.1%) reported depressive symptoms (7). Recent studies on mental health of Chinese health professionals exposed to COVID-19 found that depression (19.8–50.4%), anxiety (35.6–57.3%) insomnia (18.4–55.7%) and general distress (41.5–71.5%) were common (8–13).

Physical and mental health problems such as depression, anxiety, distress and post-traumatic stress symptoms in frontline health professionals during the COVID-19 pandemic have been frequently reported (8, 9, 13). Fatigue has been underestimated, despite it was also common in health professionals during the SARS/MERS outbreaks (7, 14). Fatigue affects the whole organism, including subjective feelings of exhaustion and deterioration of mental and physical activities (15, 16), which leads to a range of negative health outcomes such as poor morale, absenteeism, apathy, irritability, increased tendency for depression, and poor motivation at work. In addition, fatigue may also result in non-specific physical complaints such as headache, dizziness, palpitations, rapid breathing, loss of appetite, indigestion, or insomnia (17, 18).

By nature of the clinical specialty, clinicians working in ophthalmology and otolaryngology departments have a higher likelihood to be infected due to close proximity to COVID-19 patients and asymptomatic carriers compared with their counterparts in other clinical specialties. Due to the heavy workload and high risk of infection, clinicians in ophthalmology and otolaryngology departments are more likely to report physical and mental ailments including fatigue that influences quality of patient care and increase the risk of conflicts with patients. In order to reduce the occurrence of fatigue and its negative consequences, it is important to understand its patterns and associated factors in clinicians working in different specialties, particularly those working in high risk clinical settings during the COVID-19 pandemic.

This study examined the prevalence of fatigue and its association with quality of life (QOL) in clinicians working in ophthalmology or otolaryngology departments during the COVID-19 pandemic in China. The main hypothesis of the study was that fatigue would be common in these clinicians and it would be associated with lower QOL during the COVID-19 epidemic in China.

## Methods

### Setting and Sample

This cross-sectional, national online survey was conducted between March 15 and March 20, 2020. Due to the high risk of contagion, face-to-face interviews were not performed during the COVID-19 pandemic. Similar to other studies (10), snowball sampling and the WeChat-based QuestionnaireStar program were used to collect data. WeChat is a widely used social communication application with more than 1 billion Chinese users (6). With the help of the Panels of Ophthalmology and Otolaryngology of the Chinese Nursing Association, the Quick Response code (QR code) linked to the invitation to participate in the study and the assessment instruments were delivered to all panel members in each province of China by WeChat. Panel members in each province then distributed the QR Code to all hospitals in their respective areas. Clinicians in these hospitals/units participated in this study on a voluntary basis. The inclusion criteria were: (1) adults aged 18 years or above; (2) frontline clinicians (including doctors, nurses, and nurse assistants) working in ophthalmology or otolaryngology departments during the outbreak of COVID-19; and (3) ability to understand the content of assessment and provide written informed consent. Ethical approval was obtained from the Ethics Committee of Beijing Anding Hospital, China.

### Instruments

Participants' basic demographic data, including gender, age, marital status, education level, living circumstances, current smoking, type of hospital (primary/tertiary), department (ophthalmology/otolaryngology), rank [junior (nursing assistants, residents, and senior medical officers)/senior (vice consultant, consultant, and head nurses)], site of work (inpatient/outpatient), shift duty requirement (yes/no) and work experience during the 2003 SARS outbreak (yes/no), were collected. Three additional standard questions were asked: (1) whether they provided direct care to COVID-19 patients; (2) whether they had any family members, friends, or colleagues infected with COVID-19; and (3) whether there have been 500 or more local COVID-19 patients in the province where they lived/worked.

Severity of fatigue was assessed by the Numeric Rating Scale (NRS) scoring from “0”-“10,” with “0” indicating “no fatigue” and “10” indicating “unbearable fatigue” (19, 20). A total score of ≥ 4 indicated “clinically relevant fatigue (fatigue hereafter),” and ≥7 indicated “severe fatigue” (21).

Depressive symptoms were measured with the Chinese version of the Patient Health Questionnaire-9 (PHQ-9) (22, 23). The total score of PHQ-9 ranges from 0 to 27, with higher scores indicating more severe depressive symptoms (24). Global quality of life (QOL) was measured with the first two items of the World Health Organization Quality of Life Questionnaire-brief version (WHOQOL-BREF) (25, 26). Higher total scores indicate higher QOL. The Chinese version of the scale has been validated in Chinese populations with good sensitivity and specificity (27, 28).

### Data Analysis

Data were analyzed using SPSS, Version 24.0 (IBM SPSS, IBM Crop., Armonk, NY, USA). Chi-square-tests, Mann-Whitney *U*-tests, and independent samples *t*-tests compared socio-demographic and clinical characteristics between clinicians with and without fatigue, as appropriate. Multiple logistic regression analyses using the “enter” method (i.e., all independent variables were included at the same time) was conducted to examine the independent demographic and clinical correlates of fatigue. Fatigue was the dependent variable, while the variables with a *P*-value of < 0.05 in the univariate analyses were entered as independent variables. Analysis of covariance (ANCOVA) was performed to compare overall QOL between the fatigue and no fatigue groups after controlling for variables that significantly differed between the two groups in univariate analyses. The level of significance was set at *P* < 0.05 (two-sided).

## Results

In total, 3,912 clinicians met the study criteria and completed the assessments (2,155 in ophthalmology department, and 1,757 in otolaryngology department); 2,049 (52.4%; 95% CI = 50.8–53.9%) reported fatigue (NRS score ≥ 4) including 579 (14.8%; 95% CI: 13.7–15.9%) with severe fatigue (NRS score ≥ 7) during the COVID-19 outbreak. The mean NRS total score was 4.15 (SD = 2.20) in the whole sample. Table 1 shows the participants' socio-demographic and clinical characteristics.

**Table 1:** Demographic characteristics of clinicians in ophthalmology and otolaryngology departments.

**Variables**	**Total** ** (*****N*** **=** **3,912)**		**No fatigue** ** (*****N*** **=** **1,863)**		**Fatigue** ** (*****N*** **=** **2,049)**		***X*^**2**^**	**df**	***P***
	***N***	**%**	***N***	**%**	***N***	**%**			
Male gender	33	0.8	19	1.0	14	0.7	1.32	1	0.25
Married	2,929	74.9	1,369	73.5	1,560	76.1	3.65	1	0.06
College education and above	3,819	97.6	1,821	97.7	1,998	97.5	0.23	1	0.63
Living with family	3,334	85.2	1,586	85.1	1,748	85.3	0.03	1	0.88
Department							0.78	1	0.38
Ophthalmology	2,155	55.1	1,040	55.8	1,115	54.4			
Otolaryngology	1,757	44.9	823	44.2	934	45.6			
Junior clinicians	2,256	57.7	1,118	60.0	1,138	55.5	7.99	1	**0.005**
Involvement in treating SARS	428	10.9	193	10.4	235	11.5	1.23	1	0.27
Working in tertiary hospitals	3,278	83.8	1,527	82.0	1,751	85.5	8.76	1	**0.003**
Working in inpatient department	3,140	80.3	1,507	80.9	1,633	79.7	0.88	1	0.35
Shift duty	2,468	63.1	1,182	63.4	1,286	62.8	0.20	1	0.66
Local COVID-19 cases ≥ 500	610	15.6	285	15.3	325	15.9	0.24	1	0.63
Having family/friends/colleagues infected with COVID-19	208	5.3	82	4.4	126	6.1	5.92	1	**0.015**
Taking care of infected patients	299	7.6	122	6.5	177	8.6	6.04	1	**0.014**
Smoking	27	0.7	10	0.5	17	0.8	1.22	1	0.27
PHQ-9 total score ≥ 5	1,298	33.2	236	12.7	1,062	51.8	675.01	1	** <0.001**
	**Mean**	**SD**	**Mean**	**SD**	**Mean**	**SD**	**t/Z**	**df**	***P***
Age (Years)	34.2	8.1	34.0	8.4	34.5	7.9	10.52	3,910	0.054
Working experience (Years)	12.8	9.0	12.5	9.3	13.1	8.7	3.65	—^a^	** <0.001**
Total QOL score	6.6	1.5	7.3	1.3	6.0	1.5	27.72	3,910	** <0.001**

a *Mann-Whitney U-test*.

*Bolded values: < 0.05; df, degree of freedom; SD, standard deviation; COVID-19, Coronavirus Disease 2019; SARS, Severe Acute Respiratory Syndrome; PHQ-9, Patient Health Questionnaire nine items; QOL, Quality of Life*.

Univariate analyses found that fatigue was significantly associated with the clinicians' ranking, working in tertiary hospitals, having family/friends/colleagues infected with COVID-19, taking care of infected patients and PHQ-9 total score ≥ 5 (all *P*-values < 0.05). After controlling for covariates, clinicians with fatigue had significantly lower QOL compared with those without fatigue [*F*_(1, 3, 911)_ = 283.75, *P* < 0.001].

Multiple logistic regression analysis revealed that junior clinicians [Odds ratio (OR) = 0.82, 95% CI = 0.68–1.00, *P* = 0.045] had lower risk of fatigue, while working in tertiary hospitals (OR = 1.23, 95% CI = 1.02–1.49, *P* = 0.029), and presenting with more severe depressive symptoms (PHQ-9 total score ≥ 5; OR = 7.40, 95% CI = 6.29–8.70, *P* < 0.001) were factors independently associated with higher risk of fatigue (Table 2).

**Table 2:** Independent correlates of fatigue by multiple logistic regression analysis.

**Variables**	**Multiple logistic regression analysis**		
	***P-*value**	**OR**	**95% CI**
Junior clinicians	**0.045**	0.82	0.68–1.00
Working in tertiary hospitals	**0.029**	1.23	1.02–1.49
Having family/friends/colleagues infected with COVID-19	0.494	1.12	0.81–1.54
Taking care of infected patients	0.279	1.16	0.89–1.52
Working experience (years)	0.902	1.00	0.99–1.01
PHQ-9 total score ≥ 5	** <0.001**	7.40	6.29–8.70

*Bold values: < 0.05; CI, confidence interval; OR, odds ratio; PHQ-9, Patient Health Questionnaire nine items*.

## Discussion

To the best of our knowledge, this was the first study that examined the epidemiology and correlates of fatigue in clinicians in ophthalmology and otolaryngology departments during the COVID-19 pandemic. More than half (52.4%; 95% CI = 50.8–53.9%) of these clinicians suffered from fatigue (NRS score ≥ 4), and 14.8% (95% CI = 13.7–15.9%) even experienced severe fatigue (NRS score ≥ 7).

These figures (52.4%; 95% CI = 50.8–53.9%) are higher than those reported in similar studies. In a systematic review fatigue in healthcare workers during SARS/MERS/COVID-19 outbreaks was 41.2% (95% CI = 18.2–68.8%), with 80.0% (95% CI = 73.8–85.1%) during the SARS outbreak, 25.6% (95% CI = 15.5–39.3%) during the MERS outbreak and 38% (95% CI = 15.3–67.4%) during the COVID-19 epidemic (7). A survey on 140 intensive care unit nurses who volunteered to work in Wuhan from Jiangsu province (i.e., the epicenter of the COVID-19 epidemic in China) found that 15.0% reported fatigue during the COVID-19 pandemic (29). The discrepancy concerning the rate of fatigue between studies could be explained by the following reasons. First, apart from heavy clinical duties, clinicians from many ophthalmology and otolaryngology departments often had to undertake additional workload such as looking after COVID-19 patients and suspected cases during the pandemic. This led to longer shifts, less sleep, and increased psychological pressure, all of which increased the risk of fatigue (14, 30). Second, asymptomatic COVID-19 patients were more frequently encountered in hospitals than those with typical symptoms, which put clinicians at increased likelihood of being infected (31). Many clinicians stayed in hospitals, rather than returning home after work to reduce the risk of spreading virus to their family members and the wider community. This self-isolation could have further worsened their fatigue (32). In addition, during the pandemic, after looking after or contacting infected/suspected cases and patients with fever, clinicians needed to spend at least 2 weeks in quarantine, which could also lead to fatigue (9, 12, 33, 34). Fourth, various self-administered or interviewer-rating instruments on fatigue were used in studies, which could also contribute to the diversity in the findings.

Senior clinicians who were working in tertiary hospitals reported more severe depressive symptoms and were associated with higher risk of fatigue. Compare to junior clinicians, apart from clinical duties, senior clinicians, particularly those in tertiary hospitals had more administrative, teaching and academic responsibilities and pressure (13). For example, many senior clinicians were required to join the emergency response teams in their affiliated hospitals during the COVID-19 outbreak. Additionally, senior clinicians were usually older than junior colleagues, which means they might have more family responsibilities and greater financial burdens, all of which could increase the risk of fatigue. In China, clinicians working in tertiary hospitals had heavier workload and more severe burnout symptoms than those working in primary and secondary hospitals (16, 35, 36). Since January 2020, all provinces, municipalities, and autonomous regions in China have launched the first-level responses to combating the novel coronavirus disease (37). Most tertiary hospitals set up designated emergency isolation hospitals/units to provide clinical services for local infected cases (8). Many clinicians in tertiary hospitals including those working in ophthalmology and otolaryngology departments were assigned to these newly established hospitals/units, which could contribute to higher rates of fatigue.

Frontline clinicians with depressive symptoms were more likely to report fatigue in this study. The relationship between depression and fatigue was bidirectional. On one hand, clinicians who suffered from fatigue and related problems such as loss of energy, impaired concentration, irritability and reduced productivity, face a higher risk of depression (38, 39). On the other hand, fatigue is one of the common symptoms of depression (40, 41), thus there is an overlap of presentations between fatigue and depression.

The concept of QOL comprises a person's physical condition, psychological state, level of independence, social relationships, environment, and spirituality (42). Clinicians suffering from a higher level of psychological stress and burnout were at increased risk of substance abuse and suicide, particularly when they were facing huge demands at work, illness, death, interpersonal conflicts and lack of knowledge or support during a public health crisis such as the SARS/COVID-19 outbreaks (43, 44). Considering the negative impact of fatigue on the quality of clinical practice and fatigue-related negative health outcomes such as low energy, decreased physical endurance, poor cognition, and mental distress (45), it is reasonable to assume that clinicians with fatigue are more likely to have lower QOL than clinicians without it as confirmed in this study. Similar findings were also reported prior to the COVID-19 pandemic (8, 10, 13).

The strengths of this study include the large sample size, and the use of standardized instruments on fatigue and QOL. However, there were several limitations that need to be acknowledged. First, potentially important variables associated with fatigue, such as physical comorbidities, lifestyle factors (e.g., regular physical exercise and sleeping habits) and social support, were not examined due to logistical reasons. Second, the causality between fatigue and other variables could not be established because of the cross-sectional study design. Third, due to the risk of cross-infection, random sampling could not be used when recruiting frontline health professionals during the COVID-19 outbreak. Fourth, more than 95% of the participants were female clinicians, which makes the sample subject to selection bias. Finally, COVID-19 information was collected by self-report questions, thus the information obtained is likely to be affected by demographic factors, such as education level.

In conclusion, fatigue was common in ophthalmology and otolaryngology departments clinicians during COVID-19 pandemic in China. Considering the negative impact of fatigue on QOL, health authorities and policymakers should develop preventive strategies and conduct effective interventions targeting this population exposed to excessive demands in clinical practice.

## Data Availability Statement

The Ethics Committee of Beijing Anding Hospital that approved the study prohibits the authors from making the research data set publicly available. Readers and all interested researchers may contact Dr. Feng-Rong An (Email address: afrylm@sina.com) for details. Dr. An could apply to the Ethics Committee of Beijing Anding Hospital for the release of the data.

## Ethics Statement

The studies involving human participants were reviewed and approved by The Ethics Committee of Beijing Anding Hospital. The patients/participants provided electronic written informed consent to participate in this study. Electronic written informed consent was obtained from the individual(s) for the publication of any potentially identifiable images or data included in this article.

## Author Contributions

YJ, F-RA, and Y-TX: conception and design. YL and Y-TX: administrative support. X-YL and YL: provision of study materials or patients. X-YL, YL, and Y-JZ: collection and assembly of data. YJ, TC, and GU: data analysis and interpretation. YJ, Y-TX, GU, and ML: manuscript writing and revision. All authors: final approval of manuscript.

## Conflict of Interest

The authors declare that the research was conducted in the absence of any commercial or financial relationships that could be construed as a potential conflict of interest.
